# Effect of Ocular Hypertension on D-*β*-Aspartic Acid-Containing Proteins in the Retinas of Rats

**DOI:** 10.1155/2019/2431481

**Published:** 2019-05-21

**Authors:** Takashi Kanamoto, Takashi Tachibana, Yasushi Kitaoka, Toshio Hisatomi, Yasuhiro Ikeda, Yusuke Murakami, Kei Tobiume, Ryo Asaoka, Yoshiaki Kiuchi

**Affiliations:** ^1^Department of Ophthalmology, Hiroshima Memorial Hospital, 1-4-3, Honkawacho, Naka-ku, Hiroshima 730-0802, Japan; ^2^Department of Ophthalmology, Graduate School of Medical Sciences, Kyushu University, 3-1-1, Maidashi, Higashi-ku, Fukuoka 812-8582, Japan; ^3^Department of Ophthalmology, St. Marianna University School of Medicine, 2-16-1, Sugao, Miyamae-ku, Kawasaki City, Kanagawa 216-8511, Japan; ^4^Graduate School of Biomedical & Health Sciences, Hiroshima University, 1-2-3, Kasumi, Minami-ku, Hiroshima 734-8551, Japan; ^5^Department of Ophthalmology, University of Tokyo Graduate School of Medicine, 7-3-1 Hongo, Bunkyo-ku, Tokyo 113-8655, Japan; ^6^Department of Ophthalmology and Visual Sciences, Hiroshima University, 1-2-3, Kasumi, Minami-ku, Hiroshima 734-8551, Japan

## Abstract

**Purpose:**

To investigate the effect of ocular hypertension-induced isomerization of aspartic acid in retinal proteins.

**Methods:**

Adult Wistar rats with ocular hypertension were used as an experimental model. D-*β*-aspartic acid-containing proteins were isolated by SDS-PAGE and western blot with an anti-D-*β*-aspartic acid antibody and identified by liquid chromatography-mass spectrometry analysis. The concentration of ATP was measured by ELISA.

**Results:**

D-*β*-aspartic acid was expressed in a protein band at around 44.5 kDa at much higher quantities in the retinas of rats with ocular hypertension than in those of normotensive rats. The 44.5 kDa protein band was mainly composed of *α*-enolase, S-arrestin, and ATP synthase subunits *α* and *β*, in both the ocular hypertensive and normotensive retinas. Moreover, increasing intraocular pressure was correlated with increasing ATP concentrations in the retinas of rats.

**Conclusion:**

Ocular hypertension affected the expression of proteins containing D-*β*-aspartic acid, including ATP synthase subunits, and up-regulation of ATP in the retinas of rats.

## 1. Introduction

Proteins in living organisms consist exclusively of L-amino acids. However, the homochirality of amino acids is not always maintained, and D-amino acid residues have been detected in various human tissues [1]. D-aspartic acid formation is accompanied by isomerization of natural *α*-aspartic acid to abnormal *β*-aspartic acids, resulting in the presence of four isomers: the normal L-*α*-aspartic acid, plus L-*β*-aspartic acid, D-*α*-aspartic acid, and D-*β*-aspartic acid [2]. Unlike the optical characteristics, the chemical and physical properties of L-form and D-form amino acids are highly similar. Nonetheless, racemization and isomerization of amino acids in proteins have the potential to change their protein structures, and such posttranslational modifications can induce protein unfolding that contributes to certain diseases [3].

Several conditions for the inversion of the L- to the D-form of aspartic acid in living organisms have been reported. D-*β*-aspartic acid-containing proteins have been detected in the elastin of skin with dermatitis due to sun damage in elderly subjects, but not in young subjects [4]. In eyes, D-*β*-aspartic acid-containing proteins have been found in pterygia [5]. Abnormally accumulated proteins rich in AGE (N3-carboxy (methyl)-L-lysine, pyrraline, and pentosidine) and D-*β*-aspartic acid colocalize in the amyloid lesions in gelatinous drop-like corneal dystrophy [6]. Isomerization and inversion of aspartic acid residues occur in both *α*- and *β*-crystallins of the lens of elderly humans, which suggests that the inversion may disrupt crystallin function and change crystallin-crystallin interactions [7]. Indeed, the isomerization of aspartic acid residues has been shown to contribute to increases in aggregation, insolubilization, and disruption of function of proteins in the lens, leading to cataracts [8]. Drusen in patients with age-related macular degeneration have been found to be positive for D-*β*-aspartic acid [9]. The clinical utility of targeting D-aspartic acid has been examined in a mouse model of multiple sclerosis, and the results indicated that D-aspartic acid treatment had beneficial therapeutic effects [10]. In summary, D-aspartic acid-containing proteins have been detected in various diseases, including eye surface and intraocular diseases, and correlated with aging. Aging is one of the risk factors for progression in glaucoma [11].

Glaucoma is characterized by structural changes in the optic nerve head [12] and retinal ganglion cell layer [13] that result in a functional loss of the visual field [14], and ocular hypertension induces degeneration of the retina and the optic nerve through retinal ganglion cell death. Elevated intraocular pressure (IOP) induces various physiological extracellular and intracellular changes in retinal tissue [15]. Ocular hypertension blocks axonal transport in retinal ganglion cells, which leads to deficiencies in neurotrophic factors [16], and induces the oxidative stress pathway, leading to injury of the optic nerve head [17]. Oxidative stress is increased by mitochondrial dysfunction [18] and bursts of superoxide anions [19, 20]. Thus, it is strongly suspected that the pathway of oxidative stress and reactive oxygen species (ROS) formation may play important roles in the initiation and progression of glaucoma [21].

Generation of ROS accelerates the inversion rate to the D-form of aspartic acid residues, and it is indicated that oxidative stress might be closely related to D-Asp formation in aging proteins [22]. Current evidence indicates that free radicals and ROS generated by *γ*-irradiation induce oxidation of methionine and tryptophan, deamidation of asparagine and glutamine, and isomerization of aspartyl residues as well as the truncation and cross-linking of proteins [23]. Such posttranslational modification is suspected of being associated with the IOP-dependent retinal degeneration in glaucoma, but this association and the possible mechanism underlying it remain to be confirmed.

In the present study, we hypothesized that physiological intolerance to D-*β*-aspartic acid in the retina induced by ocular hypertension may contribute to the retinal degeneration in glaucoma. We showed that ocular hypertension affected the expressions of proteins containing D-*β*-aspartic acid, including adenosine triphosphate (ATP) synthase subunit *α* and subunit *β*, and that ATP concentrations were increased in the retinas of rats with ocular hypertension.

## 2. Materials and Methods

### 2.1. Animals

Wild-type adult male Wistar rats were obtained from CLEA (Tokyo) and housed in clear plastic cages containing pine bedding. The animals' quarters were kept at 21°C on a 12/12 hour light/dark cycle.

The preparation of model rats with ocular hypertension was performed as previously described [24]. Briefly, rats were anesthetized by a mixture of ketamine-xylazine (10 and 4 mg/kg, respectively) injected intracamerally with 10 *μ*l of 35% India ink (Becton Dickinson, Cockeysville, MD) in 0.01 M phosphate-buffered saline (PBS). At 1 week after the injection, a round of 200 laser beam irradiations were delivered on a dark circular band along the limbus, which was visualized as a pigmented trabecular meshwork by the aggregation of carbon particles. The argon laser settings were 200 mm diameter, 150–200 mW, for 0.2 second durations (Ultima argon laser 2000 SE; Coherent, Tokyo). At 5 weeks after the laser treatment, the IOPs of rats were measured in an awake state with a portable tonometer (Tonolab, Icare Finland, Helsinki, Finland). The eyes with ocular hypertension were defined as those having an IOP of 21 mm·Hg or greater and were enucleated.

Forty rats were treated with injection of ink and laser irradiation in the right eye, and left eyes were left untreated as controls. Four eyes had a too high IOP to be ruptured in the cornea, and 13 eyes had IOPs of 20 mm Hg or less and thus did not satisfy our definition of ocular hypertension. Finally, 23 eyes were considered suitable for modeling glaucoma, and among them, 17 were used in the experiments.

The retinas were carefully isolated from enucleated eyes in phosphate-buffered saline (PBS) and solubilized in the sample buffer (8 M urea, 4% CHAPS, 0.5% dithiothreitol (DTT), IPG buffer, and pH 3–10).

All experiments were performed in accordance with the guidelines of the Association for Research in Vision and Ophthalmology on the use of animals in ophthalmic research. The procedures used in these experiments were approved by the Animal Use Committee of Hiroshima University.

### 2.2. Western Blot Analysis

Western blot analysis was performed as previously described [25]. Briefly, lysates of the retina were subjected to 10% SDS-PAGE and the proteins were transferred to a nitrocellulose membrane, Hybond-C (GE Healthcare, Buckinghamshire, UK), and blocked with 5% milk in TBS-T buffer (10 mM Tris-HCl pH 7.4, 150 mM NaCl, 0.1% Tween-20). The membranes were incubated with a rabbit anti-D-*β*-aspartic acid antibody (CosmoBio, Tokyo) or a goat anti-actin antibody (Santa Cruz Biotechnology, Santa Cruz, CA). Horseradish peroxidase-conjugated secondary antibodies were used, and the blots were developed with enhanced chemiluminescence (ECL). Volumes of protein bands were evaluated by Multi Gauge software, Version 3.2 (Fuji Film Corp, Tokyo).

Transferred membranes were also stained with Ponceau solution (Wako, Osaka, Japan). Membranes were incubated with 0.1% Ponceau in 5% acetic acid in water for a few minutes. Then the membranes were washed with water until the background was gone and the proteins were visible.

Gels were also stained with Coomassie Brilliant Blue R-250 (Bio-Rad Laboratories, Hercules, CA). The gels were fixed in 7.5% acetic acid and 20% methanol and then stained with 0.25% Coomassie Brilliant Blue R-250. Finally, the gels were washed with 5.0% acetic acid and 7.0% methanol.

### 2.3. Enzymatic Digestion of Proteins in Gels

Selected proteins were excised from the gels and washed with 50 mM·NH_4_HCO_3_ in methanol for 20 min at 37°C and then with 100% acetonitrile for 10 min to remove the stain. The gels were reduced by 1M DTT in 100 mM·NH_4_HCO_3_ and alkylated with iso-acetoamide in 100 mM·NH_4_HCO_3_. The gels were then dried by vacuum centrifugation. The gels were rehydrated in 20 mM NH_4_HCO_3_ containing 2.5 mg/mL trypsin and incubated for 16 hours at 37°C. The supernatant was collected, and digested peptides were extracted by sonication with 0.1% trifluoro acetic acid (TFA) in 50% acetonitrile for 5 min.

### 2.4. Liquid Chromatography-Mass Spectrometry (LC-MSMS) Analysis of Tryptic Peptides

A nanoflow HPLC system was used for liquid chromatography (LC) (Paradigm MS4; Michrom Bioresources, Auburn, CA). Mass spectrometry (MS) was performed on an ion trap system (LCQ Fleet; Thermo Fisher Scientific, Waltham, MA). The peptides resulting from digestion with trypsin were separated by nanoflow HPLC using a C_18_ column (L-column, 0.1 × 150 mm; Chemicals Evaluation and Research Institute, Tokyo) with a linear gradient of 5–60% acetonitrile in the presence of 0.1% formic acid at a flow rate of 0.5 *μ*L/min over 120 min and analyzed by using Proteome Discoverer 1.0 software.

### 2.5. Measurement of ATP Concentration in the Rat Retina

Lysates of retinas derived from 8 glaucoma model rats were each diluted to 0.1 ml with PBS. Measurement of ATP was performed by enzyme-linked immunosorbent assay (ELISA) as previously described [26]. Samples were transferred into separate wells of white 96-well microplates, and the ATP levels were determined using bioluminescent enzyme-coupled assay with luciferin-luciferase reaction buffer (ATP bioluminescence assay kit, FL-AA; Sigma-Aldrich, St. Louis, MO) according to the manufacturer's recommendations. The ATP levels were detected by a luminometer (FlexStation 3 Multi-Mode Microplate Reader Molecular Devices, Sunnyvale, CA).

ATP values were normalized using natural logarithm (ln) transformation to achieve a normal Gaussian distribution, as described by Loukovaara et al. [27]. A two-sided *t*-test was used to compare the means of ln-transformed continuous variables between the two groups, and correlation coefficients were evaluated to investigate the linear relationship between the variables investigated.

## 3. Results

### 3.1. Expression of Proteins Containing D-*β*-Aspartic Acid in the Retinas of Rats with Ocular Hypertension

**Figure 1:**
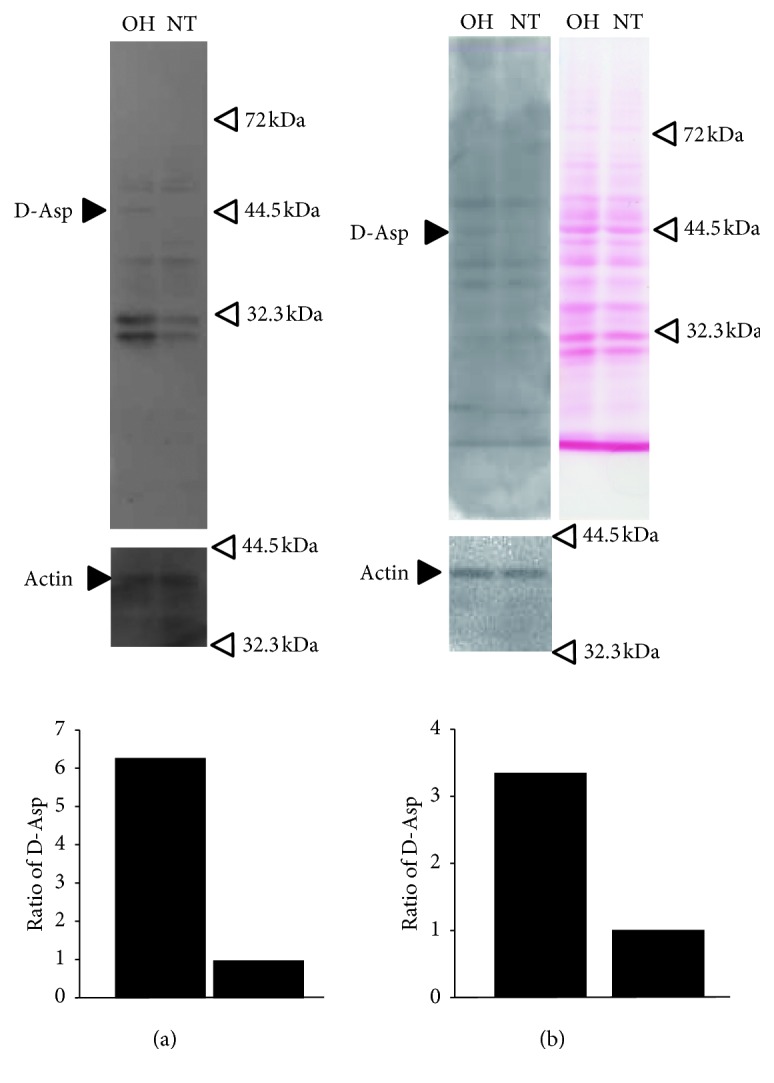
The expression of proteins containing D-aspartic acid in the retinas of rats with ocular hypertension and normotension. (a) Total lysates of retinas of rats with ocular hypertension (OH) and with normotension (NT) were applied to 10% acrylamide gel (SDS-PAGE) and blotted with anti-D-aspartic acid antibody. Blotting with anti-actin antibody was performed to standardize the total volume of applied proteins. The expression volumes of proteins containing D-aspartic acid were corrected with each actin expression, and the ratios at OH and NT are shown in the lowest panel. The black arrow, labelled D-Asp, shows a band with stronger expression in OH than NT. (b) Total lysates of retinas of OH and NT were blotted with anti-D-aspartic acid antibody (a) and stained with Ponceau solution (b).

**Figure 2:**
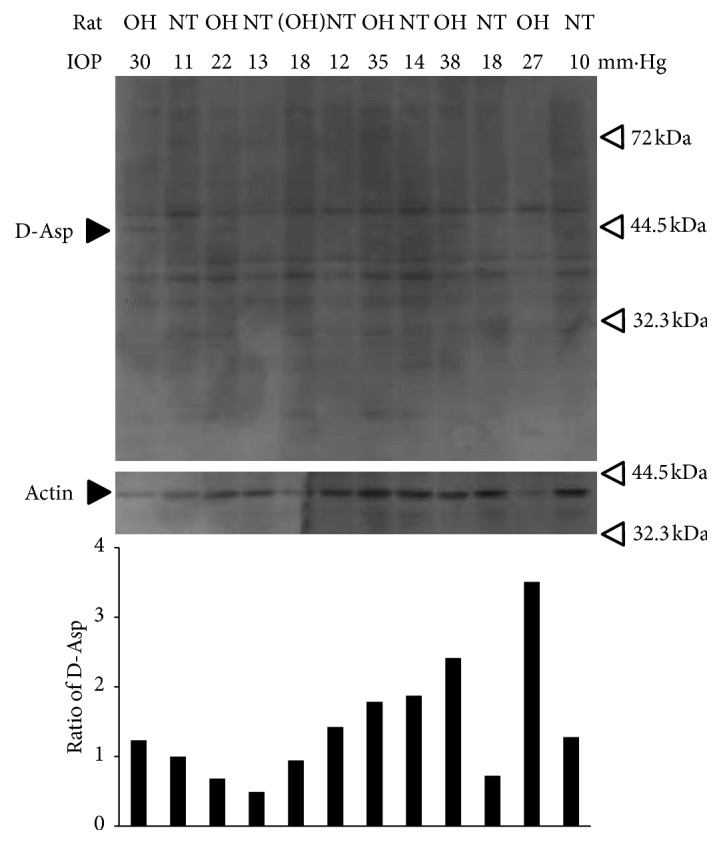
Replicability of the induction of proteins including D-aspartic acid in the retina of ocular hypertensive and normotensive rats. Total lysates of retinas derived from 6 rats with OH or NT eyes blotted with anti-D-aspartic acid antibody and anti-actin antibody. Black arrow, labelled D-Asp, shows the protein band including D-aspartic acids. The samples of the first and second lanes are the same as in Figure 1(a) and are blotted as a positive control. The fifth and sixth lanes were derived from a rat which failed to achieve glaucoma status (defined as IOP > 21) and was blotted as a negative control. Lowest panel, which shows the ratios of the expression volumes of proteins containing D-aspartic acid corrected with each actin expression, showed that protein bands in the first, third, ninth, and eleventh lane had a stronger protein band including D-aspartic acid around 44.5 kDa than control NT.

To determine whether proteins including D-*β*-aspartic acid are expressed in the retina, western blot analysis was performed. We prepared glaucoma model rats, in which the IOP in one eye was 30 mm Hg, and the IOP in the other was 11 mm Hg. Western blot analysis showed that D-*β*-aspartic acid was specifically expressed in several protein bands at around 44.5 kDa; the corresponding proteins were observed in the retinas of rats with ocular hypertension at much higher levels than in those with normal tension (Figure 1(a)). To confirm the replicability of the induction of proteins including D-*β*-aspartic acid at around 44.5 kDa, retina samples derived from other glaucoma model rats were also checked by western blot analysis, and similar results were confirmed (Figure 2).

Following the western blot analysis, we also performed Ponceau staining. The results revealed a band that appeared to represent protein with the same molecular weight and the same volume as the protein in the western blot (Figure 1(b)).

These findings indicated that the protein band around 44.5 kDa consisted of D-*β*-aspartic acid-containing proteins induced by ocular hypertension.

### 3.2. Identification of Proteins in the Protein Band including D-*β*-Aspartic Acid

**Figure 3:**
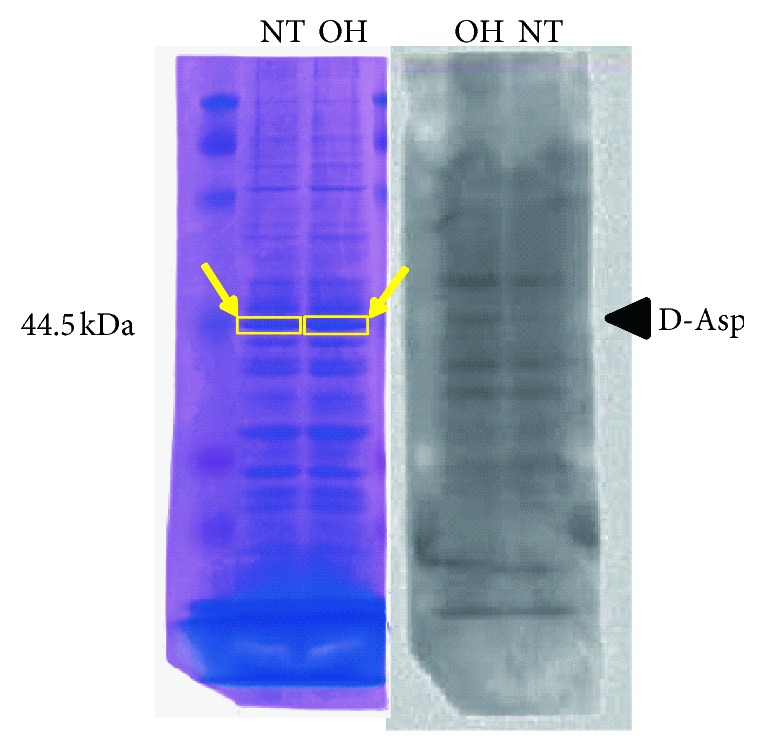
Preparation for identification of proteins including D-aspartic acid in the retinas of rats with ocular hypertension and normal tension. The target bands (yellow arrow), adjusted by western blotting (*right panel*), were cut out from gels staining with Coomassie Brilliant Blue (*left panel*) to prepare for LC-MSMS.

The band of D-*β*-aspartic acid-containing proteins in the retina with ocular hypertension was cut out from the acrylamide gel stained with Coomassie Brilliant Blue (Figure 3). The peptides were extracted from the gel by in-gel digestion with trypsin and applied to LC-MSMS to identify proteins. Because the protein band was considered to include several proteins, we selected proteins for which the probability score was higher than 30 (Table 1).

The results indicated that the band of D-*β*-aspartic acid-containing proteins around 44.5 kDa mainly included alpha-enolase, S-arrestin, glial fibrillary acidic protein, ATP synthase subunit *β*, and ATP synthase subunit *α*. Most of the identified proteins were found in both the ocular hypertensive and normotensive retinas, but the results of the western blot analysis in Figure 1 indicated that the volume of D-*β*-aspartic acid-containing proteins around 44.5 kDa was much greater in the retinas with ocular hypertension than in the normotensive retinas. Thus, it is suspected that amino acid residues isomerized to D-*β*-aspartic acid are physiologically present in the normotensive retina, but that ocular hypertension promotes further isomerization of amino acid residues.

### 3.3. Correlation of IOP and ATP Concentration in the Rat Retina

Among the identified proteins, we focused on ATP synthase because we were interested in the double identification of subunit *β* and subunit *α* in ATP synthase. Such double identification would mean that the isomerization to D-*β*-aspartic acid induced by ocular hypertension could have affected the amino acid structure of ATP synthase. To investigate the physiological change in the function of ATP synthase, we measured ATP concentrations in the retinas of rats and analyzed their correlation with changes of IOP.

**Figure 4:**
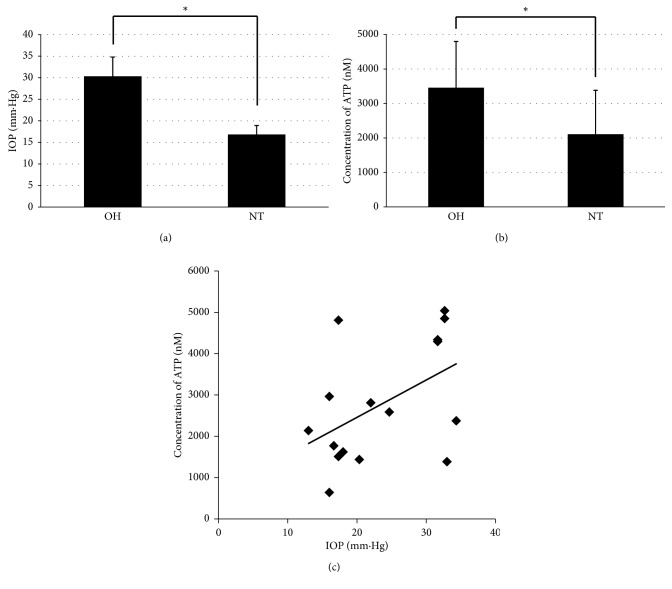
The correlation between intraocular pressure (IOP) and ATP concentration in the retinas of rats with ocular hypertension and normal tension. (a) Averages of IOPs for ocular hypertension (OH, *n*=8) and normotension (NT, *n*=8) are shown with bars representing the standard deviation (^*∗*^*p* < 0.01, *t*-test). (b) Averages of ATP concentrations in the retina are shown with bars representing the standard deviation (^*∗*^*p* < 0.01, *t*-test). (c) Intraocular pressure (IOP) and concentration of ATP of all eyes (*n*=16) were dot blotted. The line is the mean regression line.

IOPs in the group with ocular hypertension were 30.33 ± 4.45 mm·Hg (average ± standard deviation), and significantly higher than those in the group with normotension, 16.83 ± 2.08 mm·Hg (*p*=0.000016 < 0.05, *t*-test; Figure 4(a)). ATP concentrations in the retinas of the ocular hypertension group were 3457.5 ± 1339.3 nM, and significantly higher than in the group with normal IOP, 2109.2 ± 1273.2 nM (*p*=0.0291 < 0.05, *t*-test; Figure 4(b)). Moreover, the increase in IOPs was correlated with increasing ATP concentrations. The correlation coefficient between the IOP and ATP concentrations was 0.48, suggesting a positive correlation between ocular hypertension and retinal ATP concentrations (Figure 4(c)).

These results indicate that increases in ATP concentration in the retinas of rats were correlated with ocular hypertension.

## 4. Discussion

Our results showed the ocular hypertension-induced isomerization of aspartic acid in several proteins, including ATP synthase subunits; we also detected IOP-dependent up-regulation of ATP in the retinas of rats.

In addition to oxidative stress, aging is one of the factors associated with the presence of D-aspartic acid-containing proteins in living organisms [28]. While protein bands with D-aspartic acid-containing proteins were detected in both ocular hypertensive and normotensive retinas as shown in Figure 1, the band of D-aspartic acid-containing proteins in the normotensive rats may have been affected by aging so that it represents baseline expression. In ocular hypertension, the D-aspartic acid-containing proteins were increased as baseline plus ocular hypertension-induced expression. These results suggested that the D-*β*-aspartic acid-containing proteins would be affected by ocular hypertension through the oxidative stress pathway.

Aspartic residues in protein are susceptible to racemization. L-aspartic acid forms L-succinimide, which is converted to D-succinimide by keto-ethanol equilibration, leading to D-*α*- and D-*β*-aspartyl residues, and these chemical reactions are reversible. Asparagine is deamidated to L-succinimide, which hydrolyzes to L-*α*-aspartyl and L-*β*-aspartyl residues or isomerizes to D-*α* and D-*β*-aspartyl residues via D-succinimide [28]. However, in the setting of the crystalline lens, L-succinimide is inverted to D-succinimide and hydrolyzed to D-*α*- and D-*β*-aspartyl residues [29]. Hydrolysis or isomerization is accompanied by and dependent on the reaction rate constant, which is calculated by environmental factors and activation energy [30]. It is thought that ocular hypertension may play a role in isomerization as an environmental factor or activation energy.

The physiological functions of proteins are dependent on their posttranslational modification, including chemical and structural changes of amino acids. For example, the regulation of Bcl-xL deamidation plays a critical role in the tumor-specific activity of DNA-damaging antineoplastic agents [31]. In our present study, we showed that amino acid residues are isomerized to D-*β*-aspartic acid in ATP synthase subunit *β*, and this isomerization could affect the function of ATP synthase. ATP synthase subunit *α* and subunit *β* are components of F1-ATPase, which directly acts on the intracellular synthesis of ATP [32]. This suggests that dysfunction of ATP synthase may affect the function of ATPase, leading to up-regulation of ATP. To examine this hypothesis, it will be necessary to determine the location of D-*β*-aspartic acid in ATP synthase subunit *β* and to measure the enzymatic activity in F1-ATPase.

Tolerance of the ATP concentration is regulated by Muller cells, which are very important in the retina. It is because they mediate the survival of retinal ganglion cells [33]. P2Y1 receptors in Muller cells, activated by glutamate receptor, promote ATP exocytosis, and excessive ATP induces P2X7 receptor activation and up-regulates intracellular calcium ions, leading to retinal ganglion cell death [34]. These facts indicate that excessive increase of ATP in the retina with ocular hypertension may play a role in the degeneration of retinal ganglion cells in glaucoma.

Our experiment has several limitations. It is technically quite difficult to identify the type of aspartic acid residue, L-*α*-, L-*β*-, D-*α*-, or D-*β*-aspartic acid because asparagine residues in proteins can also be racemized to these four types of aspartic acid residues. Furthermore, our study did not investigate physiology directly in chronic glaucoma patients; rather, glaucoma model rats were treated for acute ocular hypertension.

In conclusion, we found that ocular hypertension affected the presence of D-*β*-aspartic acid-containing proteins including ATP synthase subunits and increased ATP concentration in the retinas of rats. We believe that our results may contribute to a better understanding of the mechanism of retinal degeneration in glaucoma because our approach was dependent on not only associated proteins but also peptides and amino acids as posttranslational modifications.

## Figures and Tables

**Table 1:** Identification of proteins.

Protein	Accession	Score	Coverage	Proteins	Unique peptide	Peptides	PSMs	Aas	MW	p*I*
*Expressed proteins identified by LC-MSMS from rat retina with ocular hypertension (OH)*										
Alpha-enolase	P04764	473.34	90.32%	3	21	50	161	434	47.1	6.57
ATP synthase subunit beta	P10719	193.82	74.67%	6	20	31	63	529	56.3	5.34
S-arrestin	P15887	135.47	79.90%	2	16	30	50	403	44.9	6.06
Glial fibrillary acidic protein	P47819	131.38	76.74%	3	20	43	58	430	49.9	5.44
ATP synthase subunit alpha	P15999	118.77	55.15%	8	15	34	49	553	59.7	9.19
Vimentin	P31000	79.57	59.44%	2	9	29	35	466	53.7	5.12
Spliceosome RNA helicase Ddx39b	Q63413	52.87	42.99%	3	7	17	22	428	49	5.67
Heterogeneous nuclear ribonucleoprotein H	Q8VHV7	42.71	40.76%	1	2	15	19	449	49.2	6.06
Dynactin subunit 2	Q6AYH5	41.95	46.27%	1	7	13	14	402	44.1	5.26
Protein kinase C and casein kinase substrate in neurons protein 1	Q9Z0W5	40.49	50.57%	1	5	20	23	441	50.4	5.24
Glutamate dehydrogenase	P10860	37.86	39.07%	1	4	17	24	558	61.4	8
Fascin	P85845	35.51	39.55%	1	4	12	14	493	54.5	6.74
Heterogeneous nuclear ribonucleoprotein H2	Q6AY09	34.5	34.74%	1	2	14	17	449	49.3	6.3

*Identified proteins in normal tension (NT)*										
Alpha-enolase	P04764	391.19	88.02%	3	19	46	140	434	47.1	6.57
ATP synthase subunit beta	P10719	177.21	71.27%	6	20	32	61	529	56.3	5.34
S-arrestin	P15887	131.58	73.95%	1	14	31	58	403	44.9	6.06
ATP synthase subunit alpha	P15999	119.11	56.78%	8	14	33	51	553	59.7	9.19
Vimentin	P31000	64.21	53.65%	3	8	26	30	466	53.7	5.12
Protein kinase C and casein kinase substrate in neurons protein 1	Q9Z0W5	63.5	56.92%	1	9	26	29	441	50.4	5.24
Glial fibrillary acidic protein	P47819	61.6	59.53%	1	9	26	29	430	49.9	5.44
Spliceosome RNA helicase Ddx39b	Q63413	52.12	51.40%	3	10	23	30	428	49	5.67
Dynactin subunit 2	Q6AYH5	48.74	52.49%	1	9	17	19	402	44.1	5.26
Glutamate dehydrogenase 1	P10860	40.67	35.13%	1	4	17	22	558	61.4	8
Heterogeneous nuclear ribonucleoprotein H	Q8VHV7	38.24	44.99%	3	5	16	21	449	49.2	6.06
4-Trimethylaminobutyraldehyde dehydrogenas	Q9JLJ3	33.23	36.64%	1	4	15	16	494	53.6	6.92

PSM: peptide sequence match; Aas: amino acids; MW: molecular weight (kDa).

## Data Availability

The data used to support the findings of this study are available from the corresponding author upon request.
